# Hit a new high

**DOI:** 10.1186/1744-8069-4-4

**Published:** 2008-01-21

**Authors:** Min Zhuo, Jianguo Gu

**Affiliations:** 1 Department of Physiology, Faculty of Medicine, University of Toronto Centre for the study of Pain, University of Toronto, 1 King's College Circle, Toronto, Ontario M5S 1A8, Canada; 2 Brain Institute and Department of Oral Surgery, Division of Neuroscience, College of Dentistry, University of Florida, Gainesville, Florida 32610, USA

## Abstract

Over the past three years Molecular Pain has grown steadily and clearly demonstrated its ability to publish novel scientific discoveries in the field of pain research in a timely fashion, hence having a strong and positive impact on pain and neuroscience research. Molecular Pain is now officially tracked by the ISI Web of Science, which allows for the calculation of its impact factor. From this calculation, we have found that Molecular Pain is now ranked at the top among pain journals in the world.

While we welcome the arrival of 2008, we are also celebrating the fourth birthday of ***Molecular Pain***. On behalf of the ***Molecular Pain ***editorial team, we would like to take this opportunity to wish a happy new year to our readers, authors, reviewers, editorial board members and staff. We also would like to thank all of you for the strong support during the past four years Your contribution and support has pushed ***Molecular Pain ***to hit a new high with an impact factor of 3.93. As the first online open access journal in pain research field, ***Molecular Pain ***has allowed novel scientific discovery in the field of pain research to be published in a timely fashion and to be accessed freely by anyone at any place in the world. This has had a strong and positive impact in the pain and neuroscience research fields.

In 2007, pain researchers and neuroscientists have witnessed the rapid and healthy growth of ***Molecular Pain***. ***Molecular Pain ***is now officially accepted and tracked by the ISI Web of Science. As a result, each citation of an article published in ***Molecular Pain ***is counted online. The inclusion of ***Molecular Pain ***online by the ISI Web of Science has also allowed us to monitor the citation of each article published in ***Molecular Pain ***as well as to calculate the impact factor of ***Molecular Pain***. Although the exact significance of the impact factor remains to be debated (see reference [1]), we believe that the impact factor is a most valued measurement of the performance of the journal. ISI Web of Science has been tracking ***Molecular Pain ***since 2006 and will announce the first official impact factor of Molecular Pain in 2009. As calculated but not yet announced officially, ***Molecular Pain ***hit an impact factor of 3.14 at the end of 2006 and 3.93 at the end of 2007. The official impact factor, to be announced next year by ISI, will most likely hit a higher score as a result of an extra year of citation tracking being included.

The online and open access has let articles published in ***Molecular Pain ***enjoy a significantly quicker citation rate in comparison with other professional journals. For example, between 2006 and 2007, the average citation rate of each article in ***Molecular Pain ***is the highest (at 1.45) among four pain journals (***Pain***, ***European Journal of Pain***, ***Journal of Pain***, ***Molecular Pain***) and two neuroscience journals (***J Neuroscience ***and ***European journal of Neuroscience***). The citation rates for other pain journals are 0.74 for ***Pain ***(the official journal for International association of Pain), 0.47 for ***Journal of Pain ***(the official journal for American Pain Society), and 0.51 for ***European Journal of Pain ***(the official journal for European Pain society). As compared with popular neuroscience journals, we are somewhat surprised to find that the ***Molecular Pain ***citation rate in 2006–2007 surpasses the ***Journal of Neuroscience ***(the official journal for American society for Neuroscience; at 0.92) and ***European Journal of Neuroscience ***(the official journal for federal association of European neuroscience; at 0.33). It should be noted, however, that the total number of papers published are different, with ***Molecular Pain ***at 66; ***Pain ***at 300; ***J of Pain ***at 120 and ***European journal of Pain ***at 94. However, for ***Molecular Pain***, the **h **factor, an index that measures the quantity of high impact papers, is ranked among the top pain journals (see Figure 1).

**Figure 1:**
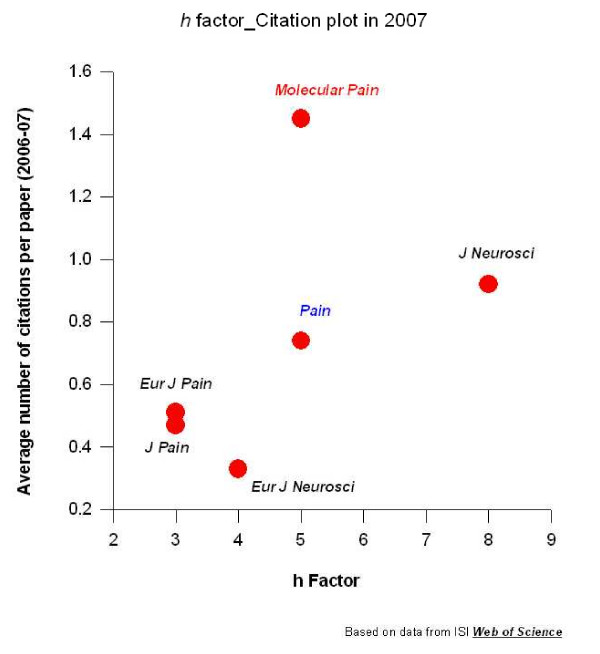
**Averaged citation rate and h factor for papers published in *Molecular Pain *and five other professional journals between 2006–2007**. The data are collected from ISI Web of Science . The meeting abstracts were excluded from the calculation. Among pain journals and two neuroscience journals, ***Molecular Pain ***has the highest citation rate for papers published between 2006 – 2007 and the **h **factor of ***Molecular Pain ***is the same as the ***Journal of Pain***.

At the beginning of 2008 and the start of the fourth year of ***Molecular Pain***, we hope that our authors, reviewers and readers will share with us the joy of this success. The ***Molecular Pain ***editorial team will continue to work hard, and together with your help, we believe that ***Molecular Pain ***will hit another new high by the end of 2008.
